# Higher Lipoprotein (a) Levels Are Associated with Better Pulmonary Function in Community-Dwelling Older People – Data from the Berlin Aging Study II

**DOI:** 10.1371/journal.pone.0139040

**Published:** 2015-09-30

**Authors:** Nikolaus Buchmann, Ursula Kassner, Kristina Norman, David Goldeck, Rahel Eckardt, Graham Pawelec, Elisabeth Steinhagen-Thiessen, Ilja Demuth

**Affiliations:** 1 Research Group on Geriatrics, Charité –Universitätsmedizin Berlin, Reinickendorfer Str. 61,13347 Berlin, Germany; 2 Lipid Clinic at the Interdisciplinary Metabolism Center, Charité-Universitätsmedizin Berlin, Augustenburger Platz 1,13353 Berlin, Germany; 3 Department of Internal Medicine II, Centre for Medical Research, University of Tübingen, Tübingen, Germany; 4 Institute of Medical and Human Genetics, Charité –Universitätsmedizin Berlin, Augustenburger Platz 1,13353 Berlin, Germany; Hunter College, UNITED STATES

## Abstract

Reduced pulmonary function and elevated serum cholesterol levels are recognized risk factors for cardiovascular disease. Currently, there is some controversy concerning relationships between cholesterol, LDL-cholesterol, HDL-cholesterol, serum triglycerides and lung function. However, most previous studies compared patients suffering from chronic obstructive pulmonary disease (COPD) with healthy controls, and only a small number examined this relationship in population-based cohorts. Moreover, lipoprotein a [Lp(a)], another lipid parameter independently associated with cardiovascular diseases, appears not to have been addressed at all in studies of lung function at the population level. Here, we determined relationships between lung function and several lipid parameters including Lp(a) in 606 older community-dwelling participants (55.1% women, 68±4 years old) from the Berlin Aging Study II (BASE-II). We found a significantly lower forced expiration volume in 1 second (FEV1) in men with low Lp(a) concentrations (t-test). This finding was further substantiated by linear regression models adjusting for known covariates, showing that these associations are statistically significant in both men and women. According to the highest adjusted model, men and women with Lp(a) levels below the 20^th^ percentile had 217.3ml and 124.2ml less FEV1 and 239.0ml and 135.2ml less FVC, respectively, compared to participants with higher Lp(a) levels. The adjusted models also suggest that the known strong correlation between pro-inflammatory parameters and lung function has only a marginal impact on the Lp(a)-pulmonary function association. Our results do not support the hypothesis that higher Lp(a) levels are responsible for the increased CVD risk in people with reduced lung function, at least not in the group of community-dwelling older people studied here.

## Introduction

Reduced lung function has been associated with cardiovascular disease (CVD) and risk of mortality; parameters of lung function have been identified as predictors of cardiovascular events, independently of age, gender or smoking habits [1–10]. In fact, CVDs are the most common cause of death in pulmonary diseases such as chronic obstructive pulmonary disease (COPD) [11]. The prevalence of CVDs in subjects suffering from lung function decline further increases in the context of metabolic syndrome, abdominal obesity and diabetes mellitus (T2D) [6,12,13].

Plasma lipoproteins have long been recognized as important factors modulating the risk of CVD [14–16]. Of these, total cholesterol and its subfractions high-density lipoprotein (HDL-cholesterol) and low-density lipoprotein (LDL-cholesterol), have been studied intensively in relation to their association with pulmonary function, mostly in the context of COPD [17–19]. The results of these studies on non-clinical cohorts, however, are somewhat contradictory. For example, Gunell and colleagues analyzed data from 2,338 study participants with a mean age of about 45 years and showed an inverse association between the forced expiratory volume in 1 second (FEV1) and total cholesterol in men and women [20].

In another study investigating 14,135 subjects in the NHANES III survey (mean age 44 ± 20 years) HDL-cholesterol and its apolipoprotein, ApoAI, were positively associated with FEV1 whereas LDL-cholesterol and its primary apolipoprotein, ApoB, were negatively associated with FEV1. The authors discuss these results in the context of adverse LDL-cholesterol effects exerted by its contribution to the endogenous oxidative burden and thereby to the pathophysiology of lung disease, in contrast to the positive effects of HDL-cholesterol with respect to immunological function and its role in preventing lung tissue damage [17]. Indeed, systemic inflammation might be the link between HDL-cholesterol, LDL-cholesterol and lung function, since the levels of the general inflammation markers C-reactive protein (CRP), interleukin-6 (IL-6) and other markers of inflammation were shown to be increased in lung disease [21]. Cholesterol is also known to be involved in inflammatory processes associated with atherogenic effects [22]. The interaction between CVD and two of its risk factors, reduced lung function and LDL-cholesterol, has been linked to systemic inflammation [22–24]. Current working hypotheses include the assumption that a systemic inflammatory process, as found in lung disease further promotes CVD [25].

Lipoprotein (a) [Lp(a)] is another lipoprotein associated independently with CVD and coronary heart disease (CHD) as well as with cardiovascular mortality [26–28]. In contrast to other lipoproteins, the serum level of Lp(a) is predominantly genetically determined with an estimated genetic contribution of more than 90% [29]. Lp(a) consists essentially of LDL-like particles covalently linked to the high molecular weight glycoprotein apolipoprotein (a) [apo (a)] via a disulphide bond [30,31]. Lp(a) is synthesized in the liver and it is suggested that the kidney plays a role in Lp(a) catabolism [32]. The physiological function of Lp(a) remains elusive.

To the best of our knowledge the relationship between Lp(a) and pulmonary function has not been investigated in the general healthy population. We therefore analyzed data from the sub-cohort of older community-dwelling Berlin Aging Study II (BASE-II) participants with respect to the association of lung function and lipid parameters, with a focus on Lp(a).

## Material and Methods

### Study participants

We analysed baseline cross-sectional data from the Berlin Aging Study II (BASE-II). This study, described earlier in detail [33], was launched to investigate mechanisms of disease development and factors associated with "healthy" and "unhealthy" aging in residents of the greater Berlin, Germany, metropolitan area by using (bio)medical, psychological, socio-economic and genetic data in a multidisciplinary approach. The BASE-II study population consists of community-dwelling elderly people (60–84 years old) and a control group of younger participants (20–36 years old). For the present study we excluded subjects younger than 60 years and participants with prevalent bronchial asthma, with a spirometric measurement not reaching the good grade quality criteria (see below) or missing Lp(a) laboratory data. This resulted in the group of 606 elderly subjects studied here. All participants gave written informed consent and the Ethics Committee of the Charité—Universitätsmedizin Berlin approved the study (approval number EA2/029/09).

### Lung function

A pre-bronchodilatator lung function test was performed using the EasyOne™ Spirometer (ndd Medizintechnik AG, Zurich, Switzerland). Spirometry was carried out according to the ATS/ERS recommendations [34]. Only spirometric measurements with a sufficient level of quality were included (minimum of two acceptable tests; difference of the two best FEV1- (forced expiratory volume in 1 second) and FVC- (forced vital capacity)-values had to be less than or equal to 200 ml). We used the FEV1/FVC ratio as an indicator of pulmonary obstruction. Predicted values of FEV1 and FVC were calculated using ECCS/ERS (European Community for Coal and Steel/European Respiratory Society) reference values [35].

### Laboratory measurements

Blood was drawn from all participants after >8 hours fasting and kept at 4–8°C until analysis on the same day. Lp(a) analyses were done on plasma using immunological turbidity tests. The other lipid parameters were measured in serum using enzymatic colorimetric tests. Thyroid-stimulating hormone (TSH) was measured using an electro-chemiluminescence immunoassay (ECLIA). TSH-levels < 0.27 mU/l and > 4.6 mU/l were determined as hyper-/hypothyreotic. Creatinine was measured by a kinetic colour test and glomerular filtration rate (GFR) was then calculated using the Cocroft-Gould approach [36]. Insulin resistance was calculated using fasting glucose and insulin levels in the homeostasis model of insulin resistance (HOMA-IR) as (fasting glucose (mg/dl) x fasting insulin (mU/mL))/405. CRP level was determined in serum samples using an immunoturbidimetric assay. Serum IL-6 and IL-10 levels were determined with the high sensitivity CBA flex kit (BD biosciences) for flow cytometry using a BD LSR-II. Performance checks with BD CS&T tracking beads ensured constant flow cytometer performance over the different measuring days. The samples containing the standards delivered together with the CBA kit were measured three times. To increase accuracy, compared to the manufacturer’s instructions an additional dilution of the standards was analysed leading to a total of nine instead of eight controls. While IL-6 and IL-10 were determined for a subsample of 91 participants (61% men, 69 ±4 years old), all other laboratory parameters were measured for all 606 subjects investigated here.

### Covariates

Body weight was measured in light clothes with a portable electronic scale to the nearest 0.1 kg and height was determined to the nearest 0.1 cm by using an electronic weighing and measuring station (seca 764, seca, Hamburg, Germany). The prevalence of type II diabetes mellitus (T2D) was determined in a first step by a survey of patient’s medical history. An oral glucose tolerance test (oGTT, 2h) was performed in case of a negative T2D history. The diagnosis of T2D was then made based on the guidelines of the European Society of Cardiology (fasting glucose > 126 mg/dl, glucose after oGTT> 200 mg/dl or HbA1c> 6.5%) [37]. Assessment of medical history also included questions on smoking status, asthma bronchiale and medication. Regular alcohol intake was assessed by questionnaire (regular alcohol intake “yes” or “no”). Participants were asked if they perform regularly physical activities (“yes” or “no”).

### Statistical Analysis

The statistical analysis was carried out using the software package SPSS 21 for Windows (IBM Inc., Chicago, USA). Lp(a) was divided into quintiles for further calculations. We used the Kolmogorov-Smirnov test to examine normal distribution of all included variables. Normally distributed variables were analyzed using the parametric Students’s t-test, variables with a skewed distribution were analyzed with Spearman correlation and the non-parametric Mann-Whitney-U-Test. Groups were compared by Chi^2^-test or Fisher’s exact test. Finally, multivariate models–with FEV1, FVC and FEV1/FVC as dependent variables–were calculated to assess the influence of low Lp(a) levels [Lp(a) quintile 1] on dependent variables, next to potential co-variables. Model 3 was recalculated including hypo-/hyperthyreoidism states (subjects with TSH-levels <0.27 mU/l were defined as hyperthyreotic and subjects with TSH-levels > 4.6 mU/l as hypothyreotic) (model 4) and including T2D (model 5) and hormone replacement therapy (model 6).

## Results

Cross-sectional data were available for 606 subjects (55.1% females, 68 [60–84] years old). The clinical characteristics of the participants are summarized in Table 1. Men were more frequently current or former smokers. Type 2 diabetes prevalence, inflammation markers, physical activities and alcohol intake did not differ significantly between men and women. GFR used as a measure of renal function, was significantly better in men (P <0.001). Lp(a) levels were significantly higher in women, however, following exclusion of subjects with GFR < 60 ml/min (n = 529; 51% women 68 (60–82) vears old) the sex difference was no longer significant (data not shown). 9,6% of the women reported current hormone replacement therapy (n = 32).

**Table 1:** Characteristics of the BASE-II participants included in the present study.

	Men (n = 270)	Women (n = 336)	p-value
Age [years]	68 (60–80)	68 (61–84)	0.385
BMI [kg/m^2^]	27.1±3.5	26.5±4.8	0.077
FEV1 [ml]	2985±592	2134±441	< 0.001
FVC [ml]	4090±681	2861±513	< 0.001
FEV1/FVC [%]	74.3 (69.5–78.1)	75.4 (71.0–78.9)	0.040
Smoking status [n;%]			
Current	33 (12)	35 (11)	
Former	146 (54)	107 (32)	< 0.001
Never	93 (34)	192 (58)	
Regular alcohol intake [n;%]	245 (90)	295 (88)	0.515
Lp(a) [mg/l]	100 (40–343)	143 (70–440)	0.012
Total cholesterol [mg/dl]	203±36	227±37	< 0.001
HDL-cholesterol [mg/dl]	56±14	71±17	< 0.001
LDL-cholesterol [mg/dl]	123±33	136±34	< 0.001
Triglycerides [mg/dl]	106 (77–143)	89 (71–119)	< 0.001
Apo A1 [g/l]	1.79±0.28	1.54±0.25	< 0.001
Apo B [g/l]	1.01±0.23	1.00±0.25	0.753
HOMA-IR	2.1±1.7	2.7±3.4	< 0.001
Antihyperlipidemic treatment [n;%]	53 (20)	46 (14)	0.097
Self-reported physical inactivity [n;%]	20 (7.5)	26 (7.8)	1.000
T2D [n;%]	41 (17)	38 (12)	0.087
TSH [mU/l]	1.75 (1.24–2.43)	1.85 (1.33–2.72)	0.258
GFR [ml/min]	84.6±17.2	75.6±18.0	< 0.001
CRP [mg/dl]	1.1 (0.6–2.1)	1.2 (0.6–2.3)	0.376
IL-6 [pg/ml]^#^	1.4 (0.8–2.2)	1.1 (0.8–1.6)	0.442
IL-10 [pg/ml]#	0.32 (0.23–0.54)	0.32 (0.17–0.43)	0.628

Results are shown as mean ±SD,median (range) or meas (interquartile range) for variables with a skewed distribution

^#^ IL-6 and IL-10 were determined for a sub-sample of 91 participants

Mean values for FEV1 and FVC were 2985±592ml and 4090±681ml in men, 2134±441ml and 2861±513ml in women.

We found a positive correlation between FEV1 and LDL-cholesterol in women. This correlation was stable even after further adjustment for age, BMI and smoking habits. The FEV1/FVC ratio showed positive correlations with LDL-cholesterol levels and total cholesterol independent of adjustment for age, BMI and smoking status (Table 2). FVC but not FEV1 or FEV1/FVC-ratio was significantly (p = 0.035, data not shown) reduced in men but not women with low cholesterol levels (< 160 mg/dl). FEV1 and FVC were clearly associated with CRP in both sexes, whereas the other pro-inflammatory marker, IL-6, which has been analysed in a subgroup of 91 study subjects, showed stronger correlations with FEV1 and FEV1/FVC only in men. No significant correlations were observed between measurements of pulmonary function and the anti-inflammatory marker IL-10 (Table 2).

**Table 2:** Partial correlation analysis between parameters of lung function and parameters of lipid metabolism and inflammation.

	Men			Women		
	FEV1	FVC	FEV1/FVC	FEV1	FVC	FEV1/FVC
Total cholesterol	.061	.079	-.017	.079	.018	.130*
HDL-cholesterol	.080	.075	.022	-.057	-.075	.013
LDL-cholesterol	.064	.073	.000	.112*	-.049	.138*
Triglycerides	-.108	-.076	-.076	.001	.018	-.005
Apo A1	.036	.033	-.008	-.047	-.085	.056
Apo B	.018	.039	-.039	.025	-.001	.044
HOMA-IR	-.012	-0.12	.012	-.066	-.105	.038
CRP	-.214*	-.239*	-.044	-.157*	-.138*	-.162*
IL-6^#^	-.404*	-.325	-.357*	-.081	-.093	.051
IL-10^#^	-.085	-.006	.190	-.004	.073	-.113

Correlation coefficients for partial correlation analysis adjusting for age, BMI and smoking status are shown

* = p < 0.05

^#^ IL-6 and IL-10 were determined for a sub-sample of 91 participants

**Fig 1:**
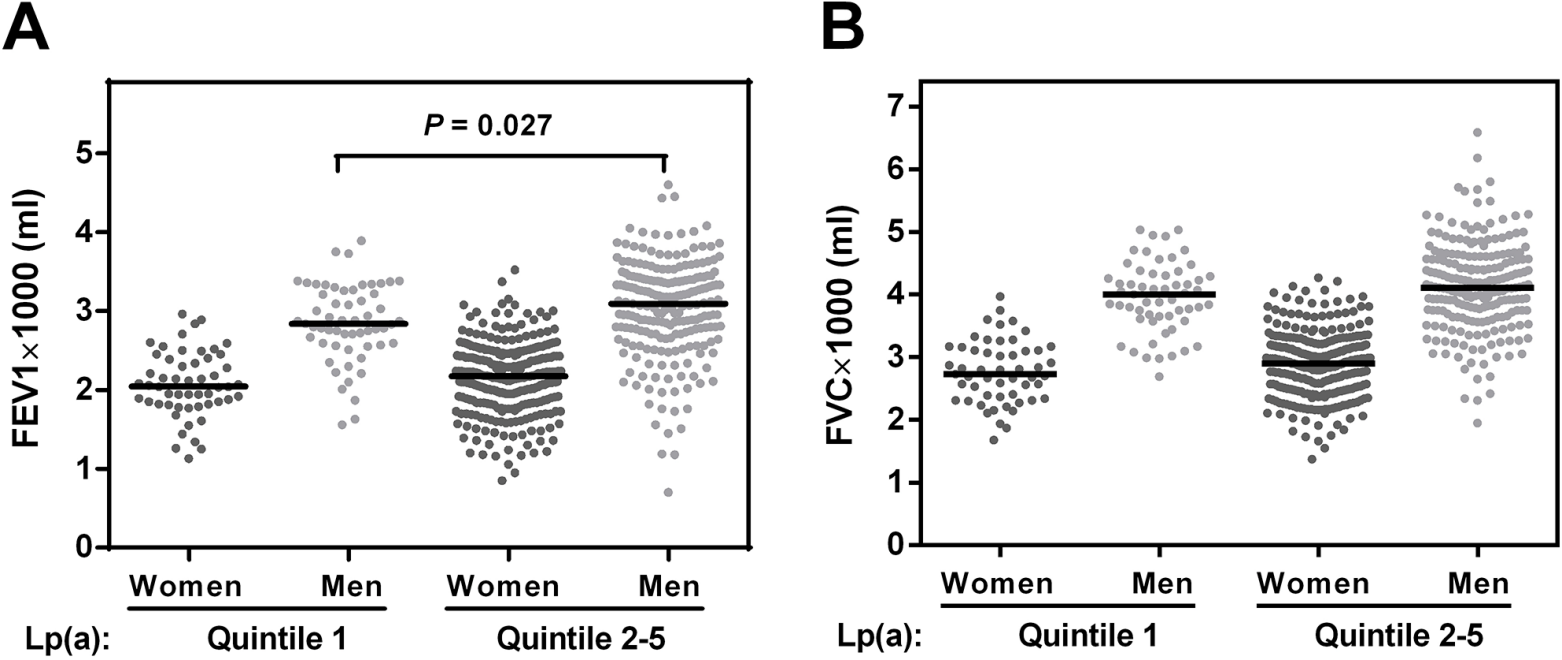
Association of pulmonary function and Lp(a) in the Berlin Aging Study II. Measurements of (A) forced expiratory volume in 1 second (FEV1) and (B) forced vital capacity (FVC) are shown separately for men and women to allow the comparison of pulmonary function from participants belonging to Lp(a) quintiles 1 with pulmonary function from participants belonging to Lp(a) quintiles 2–5. Median FEV1 and FVC (black lines) was higher in subjects from Lp(a) quintiles 2–5 in men and women. This difference was statistically significant for FEV1 in men (t-test).

Table 3 shows distributions of FEV1, FVC and FEV1/FVC according to Lp(a) quintiles. In men, FEV1 was significantly lower (t-test) in subjects within the Lp(a) quintile 1 compared to quintile 2–5 (Fig 1A). While this distribution was similar in women, the FEV1 difference in the two groups did not reach statistical significance (P = 0.231). FVC did not show statistically significant differences (t-test) in the two groups compared, i.e. Lp(a) quintile 1 vs. Lp(a) quintiles 2–5 (Fig 1B).

**Table 3:** Characteristics of the study population according to Lp(a) quintile.

	Men (n = 270)		p-value	Women (n = 336)		p-value
	Lp (a)-Quintile 1	Lp (a)-Quintile 2–5		Lp (a)-Quintile 1	Lp (a)-Quintile 2–5	
Age [years]	67±3	69±4	0.077	67±3	68±4	0.087
BMI [kg/m^2^]	27.4±3.9	27.0±3.4	0.442	27.1±4.6	26.4±4.8	0.287
FEV1 [ml]	2849±494	3021±612	0.027	2069±416	2147±445	0.231
FVC [ml]	3955±560	4125±706	0.055	2769±502	2878±514	0.134
FEV1/FVC [%]	73 (68–77)	75 (70–79)	0.091	76 (72–79)	75 (71–79)	0.536
CRP [mg/dl]	1.2 (0.5–2.3)	1.1 (0.6–2.0)	0.525	1.1 (0.6–2.2)	1.2 (0.7–2.2)	0.539
IL-6 [pg/ml] ^#^	1.0 (0.6–2–2)	1.4 (0.9–2.2)	0.483	0.92 (0.62–0.92	1.3 (0.8–1.7)	0.142
IL-10 [pg/ml] ^#^	0.30 (0.27–0.49)	0.32 (0.22–0.57)	0.825	0.08 (0.06–0.33)	0.32 (0.22–0.43)	0.166
HOMA-IR	2.0±1.4	2.6±2.8	0.345	2.7±3.7	2.9±2.2	0.220
TSH [mU/l]	1.7 (1.2–2.4)	1.8 (1.3–2.4)	0.745	1.7 (1.2–2.4)	1.9 (1.4–2.8)	0.114
GFR [ml/min]	89.2±15.7	83.4±17.4	0.026	79.5±19.1	74.8±17.7	0.108
Self-reported physical inactivity [n;%]	3 (5)	17 (8)	0.581	8 (15)	18 (6)	0.046
T2D [n;%]	15 (30)	26 (14)	0.020	10 (21)	28 (11)	0.052

Results are shown as mean ±SD or median (interquartile range) for variables with a skewed distribution

^#^ IL-6 and IL-10 were determined for a sub-sample of 91 participants

Other lipid parameters, inflammation markers, smoking status, alcohol intake and lipid lowering medication did not differ significantly within the Lp(a) quintiles. However, T2D occured more frequently in Lp(a) quintile 1 when compared to Lp(a) quintiles 2–5, notably in men (p = 0.020). Physical inactivity was more frequently reported in women within Lp(a)-Quintile 1.

To assess the association of low Lp(a) [Lp(a) quintile 1] to lung volumes (FEV1, FVC and FEV1/FVC-ratio) we further calculated linear regression models as shown in Table 4. Model 1 was simply adjusted for age and BMI. In model 2 smoking status, alcohol intake and physical activity, as well as HOMA-IR as a marker for insulin resistance were added. Further, CRP, renal function (GFR) and thyroid function (TSH) were included (model 3). Both men and women in Lp(a) quintile 1 showed significantly reduced FEV1 and FVC in adjusted model 3. FEV1 was reduced in men within the Lp(a)-quintile 1 compared to quintiles 2–5 by 228.6ml and by 132.3ml in women, FVC by 238.9ml in men and by 150.8ml in women, respectively. Distribution of FEV1/FVC, however, did not depend on Lp(a) quintiles. In addition to Lp(a) quintile 1, age and CRP levels were associated with FEV1 and FVC, independent of sex. IL-6 and IL-10 have not been included in the calculated models because these parameters were only available for a subgroup of 91 subjects (61% men, 69 ±4 years old).

**Table 4:** Multivariate models to assess the influence of low Lp(a) levels [Lp(a) quintile 1] on lung volumes.

	Men									Women								
Lp(a) quintile 1	FEV1			FVC			FEV1/FVC			FEV1			FVC			FEV1/FVC		
	Beta	SE	p	Beta	SE	p	Beta	SE	p	Beta	SE	p	Beta	SE	p	Beta	SE	p
Model 1	-207.8	86.2	.017	-210.0	94.5	.027	-1.4	1.3	.292	-105.0	62.1	.092	-131.6	71.9	.068	0.02	1.3	.987
Model 2	-227.6	87.3	.010	-218.5	96.7	.025	-1.7	1.4	.208	-110.1	63.3	.083	-130.0	73.8	.079	-0.4	1.3	.780
Model 3	-228.6	87.9	.010	-238.9	95.2	.013	-1.4	1.4	.314	-132.3	62.7	.036	-150.8	73.4	.041	-0.5	1.3	.707
Model 4	-227.3	88.1	.011	-237.1	95.4	.014	-1.4	1.4	.321	-132.8	62.8	.035	-151.9	73.2	.039	-0.5	1.3	.703
Model 5	-217.7	89.6	.016	-212.8	96.6	.029	-1.6	1.4	.258	-127.4	62.9	.044	-142.6	73.4	.053	-0.6	1.3	.676
Model 6	-213.7	89.8	.018	-239.0	96.2	.035	-1,7	1,4	.241	-124.3	62.1	.046	-135.2	72.5	.063	-0.6	1.3	.645

Model 1: adjusted for age and BMI

Model 2: Model 1 + alcohol intake, physical activity, smoking habits, HOMA-IR

Model 3: Model 2 + CRP, TSH and GFR

Model 4: Model 3 + Hyper/Hypothyreodism

Model 5: Model 3 + T2D

Model 6: Model 5 + Hyper/Hypothyreodism + hormone replacement therapy (women)

Further adjustment for hypo- and hyperthyreoidism (model 4), T2D (model 5) and hormone replacement therapy (model 6) only had a marginal impact on the Lp(a) association with lung function. As expected, BMI and smoking habits had a statistically relevant impact on FEV1 and FVC. Other parameters included in these models did not show a clinical or statistically relevant influence on lung function parameters (data not shown).

Recalculation of models 1 to 3 using logarithmic Lp(a) values and FEV1, FVC and FEV1/FVC as dichotomous variables (sex specific tercentiles, see S1 File) further supported our finding of the association between higher logarithmic Lp(a) levels and better lung function (Table A in S1 File).

## Discussion

In the present study, we investigated pulmonary function in the BASE-II sub-cohort of community-dwelling older participants with respect to plasma lipid profiles. Lp(a) values were significantly higher in women, which is in accordance with the fact that Lp(a) levels increase after menopause. Another known factor influencing the Lp(a) serum concentration is the glomerular filtration rate (GFR), which is lower in BASE-II women and thereby might additionally explain the observed Lp(a) sex difference, as indicated by our analysis excluding subjects with GFR < 60 ml/min [38]. Partial correlation analysis adjusting for age, BMI and smoking status revealed positive associations of total cholesterol with FEV1/FVC, LDL-cholesterol with FEV1 and LDL-cholesterol with FEV1/FVC which were only seen in women. No correlations were observed with respect to the apolipoproteins, Apo A1 and Apo B, and triglycerides.

The main finding of the current study is the positive association of measures of pulmonary function, FEV1 and FVC, with Lp(a) in BASE-II participants. This result became apparent for men in group comparisons and was seen in both sexes after adjusting for other variables potentially affecting lung function and/or Lp(a) serum concentration in the regression models (models 1–4). Among the factors considered in model 3 are TSH and GFR, to control, at least in part, for thyroid and renal function, which have been shown to modulate the Lp(a) level [38], whereas the age of the participants, their physical activity and alcohol intake were considered because of their known impact on pulmonary function. Inclusion of hypo- and hyperthyroidism as a covariate (model 4) did not change these results significantly. Diabetes is another possible confounding factor as Lp(a) levels have been shown to be lower in diabetic patients [38]. This was also true for the participants of BASE-II studied here. A diabetic state is known to be associated with reduced lung function [39]. Including T2D in our analysis (model 5), however, only had a marginal impact on the association of Lp(a) and lung function in men. In women the FVC-Lp(a) association was no longer seen after adjustment for T2D, whereas the FEV1-Lp(a) association did not change significantly. This does not rule out that pathophysiological factors in the context of T2D might contribute to the Lp(a)-lung function association identified here. Further, adjustment for hormone replacement therapy, which is known to lower Lp(a)-levels, did not change these results. Recalculating model 1 to 3 with logarithmic Lp(a) values and dichotomous values for FEV1, FVC and FEV1/FVC added further support for our result of the positive association between Lp(a) and lung function.

While the relationship between Lp(a) and lung function has not been studied before in a population-based cohort, it has been addressed in a clinical setting by two studies. One of these compared the lipid profile of 90 COPD patients and a control group of 90 healthy subjects and found significantly higher Lp(a) levels in the latter. Thus our data extend this earlier finding of a Lp(a)-lung function association identified in a clinical COPD cohort to the general population, at least to the group of older community-dwelling people analyzed here. The authors of the study on the COPD cohort speculated that moderate liver failure in COPD patients might impact on Lp(a) levels, since the liver is the exclusive site of Lp(a) synthesis [40]. This, however, cannot explain the association of Lp(a) with lung function in our generally healthy cohort. The other study compared only 20 COPD cases with 20 controls and found no difference in Lp(a) concentrations between the two groups [41]. Considering the extremely skewed distribution of Lp(a), the latter study investigating only 20 individuals in each group is certainly underpowered and thereby not suited to detect reliable differences.

CRP and IL-6 were included in our partial correlation analysis as markers of general inflammation processes and showed strong inverse correlations with parameters of pulmonary function in men and women. Including CRP in model 3 indicated, however, that it only has a marginal effect on the Lp(a)-lung parameter associations. Chronic inflammation is a major feature of COPD and the association between functional lung parameters and serum markers of chronic inflammation is well established in this clinical context [21]. Similar to our results, the few available population-based cohort studies which investigated the relationship between CRP and lung function also observed higher CRP to be associated with reduced functional pulmonary parameters [17,23,42–44]. Longitudinal data from Shaban and colleagues suggest that CRP might be predictive for declining lung function [42]. Two other longitudinal studies on this issue yielded discrepant results [44,45]. While lung function parameters were highly correlated with the proinflammatory status as determined by CRP and IL-6 in our study, we found no difference between the CRP and IL-6 mean levels when comparing subjects with lower vs. higher Lp(a) serum concentrations. These results suggest that increased inflammatory parameters cannot explain reduced Lp(a) levels and are in line with earlier observations on another Caucasian cohort [46].

Although there is no obvious explanation for the relationship of Lp(a) and lung function as observed in the current study, it may be hypothesized that Lp(a) has a so far unknown role in lung function which is negatively affected by lower Lp(a) levels. Whether an unidentified common cause may underlie the Lp(a)-lung function association found here remains unclear. Additionally, a role of insulin resistance and diabetes has not been ruled out completely by our analysis and should be investigated in an independent and larger cohort. The initial idea, however, that higher levels of Lp(a) might be responsible for the increased risk of developing CVDs in subjects with reduced lung function is not supported by our results. There is a need for additional studies with longitudinal approaches to confirm these results and to provide insight into the physiological relationship between lung function and Lp(a).

## Supporting Information

S1 File (DOCX)
