# Supplementary material for: Higher Lipoprotein (a) Levels Are Associated with Better Pulmonary Function in Community-Dwelling Older People – Data from the Berlin Aging Study II
Source: PLoS One. 2015 Sep 30;10(9):e0139040. doi: 10.1371/journal.pone.0139040 (PMC4589348; doi:10.1371/journal.pone.0139040)
Supplement: S1 File — (DOCX) [file pone.0139040.s001.docx]

**S1 File. Multivariate models to assess the influence of logarithmic Lp(a) levels on FEV1, FVC and FEV1/FVC [tercentile 1].**

**Table A.**

| Log Lp(a) | FEV1^ | | | FVC^ | | | FEV1/FVC^ | | |
| --- | --- | --- | --- | --- | --- | --- | --- | --- | --- |
|  | Beta | SE | p | Beta | SE | p | Beta | SE | p |
| Model 1 | 0.035 | 0.013 | .010 | 0.026 | 0.013 | .052 | -0.002 | 0.014 | .876 |
| Model 2 | 0.039 | 0.014 | .005 | 0.03 | 0.013 | .027 | 0.001 | 0.014 | .956 |
| Model 3 | 0.038 | 0.014 | .005 | 0.03 | 0.014 | .026 | -0.001 | 0.014 | .931 |

^ sex specific tercentile 1 vs. tercentile 2 and 3 for FEV1, FVC and FEV1/FVC

Model 1: adjusted for age and BMI

Model 2: Model 1 + alcohol intake, physical activity, smoking habits, HOMA-IR

Model 3: Model 2 + CRP, TSH and GFR
